# A Broken Breast

**Published:** 1889-12

**Authors:** Frank Kraft

**Affiliations:** Sylvania, Ohio


### A BROKEN BREAST.

Frank Kraft, M. D., Sylvania, Ohio.

I expect nothing less than that there will be an indignant rising *en masse* of the scientific homœopaths, when I admit, as I am constrained to do in the interest of truth, that I made use, and successfully, of Lac caninum in this case of broken breasts which I propose relating. But *magna est veritas*, etc.

On Monday, November 4th, I was asked to visit a young woman living on the southern border of Michigan, who had, five weeks before, been brought to bed with a healthy nine-pound girl (*primipara*), all without medical aid. Owing, however, to the difficulty experienced by the infant in getting any breastmilk, attributed to a tongue-tie, a bottle with the usual long rubber tube had been substituted flanked by a bottle of good old Mother

Winslow's Soothing Syrup. It goes almost without saying that in a short time the breast became filled and needed to be drawn. For this purpose, one of the exquisite torturing engines of the Inquisition, to wit, a breast-pump with a flaring mouth like unto the ancient blunderbuss and rubber bulb was applied, and with the usual result of setting up inflammation from the frequent bruising. The breasts grew tender, then hard, then painful. A doctor was called in, a most potent, grave, and reverend signior of the Old School, who succeeded in short order in adding still more to the poor woman's suffering by the use of Morphine, which effectually closed the bowels, deranged the urinary function, coated the tongue, confused her mind, yet, withal, not alleviating her sufferings. After ten days' experimenting of this kind, the parents of the patient, seeing unmistakably that she was sinking, dismissed the Old School Æsculapius and I arrived on the scene. To detail the numerous devices resorted to by his Oldschoolship, the many salves, greases, lotions, and liniments used, many of which still littering the commode, would be needless waste of time, because an old story to every physician who has ever followed the allopath. I found each breast covered with a generous-sized pancake. Removing these, there was disclosed a fistulous opening in the right breast, discharging sluggishly a greenish-yellowish-bloody pus, exceedingly offensive, and corroding the surfaces if suffered to remain for half an hour. The breast was large, hard, hot, and of a mahogany color. The left breast was also hard, hot, and brown, but not yet ready to "point," though in one spot there was a suspicious feel of softness. Otherwise the history was: cold feet, cold hands, dull headache, "back broke in two," constipation, urine scant, thick, and brown; no appetite, a little sip of cold water occasionally, which was grateful; no sleep for five nights, color of face and body a transparent, sickly pallor; clammy sweat here and there about the body, eyes dull and deep-sunken, unable to move hand or foot save with great pain, lips colorless, and the dreaded white line outlining the gums, pulse feeble, barely to be counted.

Had these symptoms presented at the first visit of any doctor, especially a homœopath, some considerable study of the repertories would have been necessary. But following an Old School drug-store, there was nothing for it but to follow Hering's advice and antidote the drugs, which was promptly done with Nux, hoping thereby to separate the drug disease from the disease of the patient. I at once gave the breasts a soap-and-water bath, then a bathing with hot water to which a few drops of Phytolacca had been added, and a cloth saturated in the same solution kept on the breast to be renewed hot every two hours as long as patient was awake. I remained a number of hours, but at last, not yet being very clear as to the remedy, I gave Phytolacca in broken doses.

On returning the following day a decided change for the better had ensued. The patient had slept a number of hours, had called for a little to eat, mainly sours and cold, the right breast had decreased in size, the mahogany color was almost all gone, the abscess was discharging laudable pus, and handling was borne better. The left breast, however, looked more angry; the swelling was much more painful, the slightest jar was unbearable, there was an unmistakable increase in suffering toward supper time, and the weight of the gland was torture. My teaching had been, not to repeat so long as improvement was manifest; the right breast was better in every way, as were also the general symptoms. I did not wish to leave the patient for twenty-four hours with that painful left breast, notwithstanding the general improvement. Still a careful study of the case impelled me to give Sac. lac., and advise the Phytolacca lotion as before.

During this ensuing night, however, she had not slept because of the pain in the left breast; the right one also had begun again to be troublesome. I went to my buggy and brought in such of my library as I had with me, and in rummaging in my overcoat pocket to my great joy found the July, 1889, copy of THE HOMŒOPATHIC PHYSICIAN, which I had been reading some days before while riding through the country. Here I remembered to have seen Brother Guernsey's "Mastitis," and if ever a drowning man grasped at a straw, it was this writer, as he hurriedly turned the leaves and found the "Mastitis" article. In a few minutes that patient had a dose of Lac caninum on her tongue. And from that moment I date the gradual recovery of my patient. The breast broke superficially, discharged a little laudable pus, the swelling went down rapidly, the color faded out, sleep came and refreshed her, appetite also returned, and with the exception of one dose of Silicea given at the last because of the peculiar difficulty in stooling, and strapping the breasts for support, she received no other medicine, and I discharged myself on the 12th with profuse thanks and encomia—and my fee.

The patient was sitting in her chair "changing" her baby, and feeling strong when I last saw her. The fistulæ had almost closed, cicatrices were forming, and the milk, which I had ordered drawn during all this period with a hot bottle, was filling the glands again, with every prospect of soon applying the child to the breast.

